# Complete Genome Sequence of a Bacterium Representing a Deep Uncultivated Lineage within the *Gammaproteobacteria* Associated with the Degradation of Polycyclic Aromatic Hydrocarbons

**DOI:** 10.1128/genomeA.01086-16

**Published:** 2016-10-06

**Authors:** David R. Singleton, Allison N. Dickey, Elizabeth H. Scholl, Fred A. Wright, Michael D. Aitken

**Affiliations:** a Department of Environmental Sciences and Engineering, University of North Carolina, Gillings School of Global Public Health, Chapel Hill, North Carolina, USA; b Bioinformatics Research Center, North Carolina State University, Raleigh, North Carolina, USA

## Abstract

The bacterial strain TR3.2, representing a novel deeply branching lineage within the *Gammaproteobacteria*, was isolated and its genome sequenced. This isolate is the first cultivated representative of the previously described “Pyrene Group 2” (PG2) and represents a variety of environmental sequences primarily associated with petrochemical contamination and aromatic hydrocarbon degradation.

## GENOME ANNOUNCEMENT

In a prior stable isotope probing (SIP) study to determine bacterial degraders of the high-molecular-weight polycyclic aromatic hydrocarbon (HMW-PAH) pyrene in an aerobic bioreactor treating contaminated soil, we identified a cluster of deeply rooted gammaproteobacterial 16S rRNA gene sequences that we designated “Pyrene Group 2” (PG2) (1). Subsequent SIP experiments in our group with the PAHs phenanthrene, fluoranthene, and benz[*a*]anthracene, as well as with pyrene in additional soils, further associated organisms within PG2 with PAH removal (2–4). The described organisms most similar to PG2 sequences were species within the *Thioalkalivibrio* and *Methylococcus* genera (~91% 16S rRNA gene similarity). However, a variety of environmental sequences primarily derived from studies investigating oil- and PAH-polluted soils and sediments (5–9) displayed much higher similarities (~99%).

Strain TR3.2 was isolated from PAH-contaminated soil collected from the site of a former manufactured gas plant in Salisbury, NC, USA that had been treated in a lab-scale, slurry-phase, aerobic bioreactor. DNA was extracted from pure cultures of strain TR3.2 using the Wizard Genomic DNA purification kit (Promega, Madison, WI, USA). The PacBio RSII platform utilizing a total of 4 single-molecule real-time (SMRT) cells over two separate library preparations was used to acquire the raw sequence reads at the University of North Carolina High-Throughput Sequencing Facility. The reads were *de novo* assembled into a single contig with SMRT Analysis 2.2 using HGAP3 and the contig was polished using Quiver (10). Significant overlap between the ends indicated that the molecule was circular and the minimus2 assembler was used to circularize a split contig (11). Final polishing was performed with Quiver and the resulting consensus sequence had an accuracy of >99.999%. The final assembly was a single, circular chromosome comprising 3,243,537 bp with an average coverage of 467×. The genome was annotated by both the Department of Energy’s Joint Genomics Institute Integrated Microbial Genomes (JGI-IMG) system and the NCBI Prokaryotic Genome Annotation Pipeline. The JGI-IMG annotation predicted 3,114 genes, of which 3,053 were predicted to encode proteins. Analysis of the genome using the software RNAmmer (12) identified only a single copy of each of the rRNA genes. The 16S rRNA gene of TR3.2 was most closely related to two environmental clones recovered during SIP experiments with PAHs in our lab (99.8% similarity). The mol% G+C of the genome was 67.79%.

The genome of TR3.2 was analyzed using AromaDeg (13) to detect genes putatively associated with degradation of aromatic compounds. Two novel genes were identified likely to encode for the large subunit of ring-hydroxylating dioxygenases (RHD), enzymes which catalyze the initial reaction in the aerobic metabolism of many PAHs. RHD gene sequences highly similar to those predicted in TR3.2 (>93% amino acid identity) have been independently recovered in separate studies analyzing functional genes in microbial communities of petroleum-contaminated soils (14, 15) and may have been derived from uncultivated bacteria closely related to TR3.2.

Strain TR3.2 is currently being characterized as the type strain of a genus representing a novel lineage within the *Gammaproteobacteria*.

### Accession number(s).

The NCBI-annotated genome of TR3.2 was deposited in GenBank under the accession number CP014671. The JGI-annotated version is available at the DOE JGI-IMG website under the name “Gammaproteobacterium strain TR3.2.”
